# Profiling of subcellular EGFR interactome reveals hnRNP A3 modulates nuclear EGFR localization

**DOI:** 10.1038/s41389-020-0225-0

**Published:** 2020-04-22

**Authors:** Tong-Hong Wang, Chih-Ching Wu, Kuo-Yen Huang, Wen-Yu Chuang, Chuen Hsueh, Hsin-Jung Li, Chi-Yuan Chen

**Affiliations:** 1 Graduate Institute of Health Industry Technology and Research Center for Food and Cosmetic Safety, Research Center for Chinese Herbal Medicine, College of Human Ecology, Chang Gung University of Science and Technology, Taoyuan, 333 Taiwan; 2 Tissue Bank, Chang Gung Memorial Hospital at Linkou, Taoyuan, 333 Taiwan; 3 Department of Otolaryngology-Head & Neck Surgery, Chang Gung Memorial Hospital at Linkou, Taoyuan, 333 Taiwan; 4 Department of Medical Biotechnology and Laboratory Science, College of Medicine, Chang Gung University, Taoyuan, 333 Taiwan; 5 Molecular Medicine Research Center, Chang Gung University, Taoyuan, 333 Taiwan; 6 Research Center for Emerging Viral Infections, College of Medicine, Chang Gung University, Taoyuan, 333 Taiwan; 7 Institute of Biomedical Sciences, Academia Sinica, Taipei, 115 Taiwan; 8 Department of Pathology, Chang Gung Memorial Hospital at Linkou and Chang Gung University, Taoyuan, 333 Taiwan; 9 Institute of Cellular and Organismic Biology, Academia Sinica, Taipei, 115 Taiwan

**Keywords:** Cancer, Drug discovery

## Abstract

The aberrant subcellular translocation and distribution of epidermal growth factor receptor (EGFR) represent a major yet currently underappreciated cancer development mechanism in non-small cell lung cancer (NSCLC). In this study, we investigated the subcellular interactome of EGFR by using a spectral counting-based approach combined with liquid chromatography–tandem mass spectrometry to understand the associated protein networks involved in the tumorigenesis of NSCLC. A total of 54, 77, and 63 EGFR-interacting proteins were identified specifically in the cytosolic, mitochondrial, and nuclear fractions from a NSCLC cell line, respectively. Pathway analyses of these proteins using the KEGG database shown that the EGFR-interacting proteins of the cytosol and nucleus are involved in the ribosome and spliceosome pathways, respectively, while those of the mitochondria are involved in metabolizing propanoate, fatty acid, valine, leucine, and isoleucine. A selected nuclear EGFR-interacting protein, hnRNP A3, was found to modulate the accumulation of nuclear EGFR. Downregulation of hnRNP A3 reduced the nuclear accumulation of EGFR, and this was accompanied by reduced tumor growth ability in vitro and in vivo. These results indicate that variations in the subcellular translocation and distribution of EGFR within NSCLC cells could affect tumor progression.

## Introduction

The epidermal growth factor receptor (EGFR) signaling is one of the most commonly deregulated pathways in human tumor. Despite the firmly-established significance of this pathway in tumor growth, however, targeted treatment aimed to disrupt EGFR has yielded only modest medical success in the past 2 decades. An exception is non-small cell lung cancer (NSCLC) patients carrying EGFR activation mutations: such patients initially showed very promising responses to treatment with an EGFR kinase inhibitor, but almost all of the treated patients eventually developed resistance to the EGFR kinase inhibitor^1^. These unsatisfactory effects are, in part, due to the highly complex of the EGFR network pathway.

Significant research efforts have sought to gain deeper information of the EGFR signaling and EGFR-mediated fatal oncology in human cancer. Recent studies have shown that activated EGFR may escape lysosome-mediated degradation and recycle to the plasma membrane or undergo intracellular trafficking to subcellular organelles, such as nuclei^2,3^ and mitochondria^4,5^. Within these organelles, EGFR may exert novel functions that differ from its typical function as a transmembrane receptor tyrosine kinase. In support of this view, the functionality of EGFR has been shown to depend on its subcellular location^6^, and EGFR was shown to undergo shuttling into the cell nucleus and mitochondrion upon ligand binding, EGFR-targeted therapy, and other stimuli (e.g., radiation)^7^. As the EGFR localized in these organelles can display novel functions and may regulate the response of a tumor to therapy, it is important to characterize the novel functions of EGFR in these organelles.

The presence of full-length EGFR in the nucleus has been recognized for over 20 years^8^. Nuclear EGFR performs as a tyrosine kinase, transcriptional mediator, and regulator of other biological functions. Within the cell nucleus, EGFR roles as a transcriptional mediator through its specific transactivation domain^9^ and through its connections with RNA helicase A^10^ and/or DNA-binding transcription factors that are highly presented in tumors, including STAT3^2^, E2F1^11^, and STAT5^12^. The nuclear increase of EGFR has been associated with cancer malignancy, poor patient survival, and drug resistance^3,13,14^. In line with these links, studies have shown that nuclear EGFR activates the expression of cyclin D1^9^, inducible nitric oxide synthase^2^, B-Myb^11^, COX-2^15^, aurora A^12^, c-Myc^16^, and breast cancer resistance protein^17^. Nuclear EGFR also keeps its tyrosine kinase activity and phosphorylates proliferating cell nuclear antigen to stimulate cell growth and DNA repair^18^. Heterogeneous nuclear ribonucleoprotein A3 (hnRNP A3) has been reported to interact with nuclear EGFR and stabilize mRNAs involved in aerobic glycolysis in response to irradiation^19^. Overall, the current evidence suggests that blocking the nuclear functions of EGFR may maximize the efficacy of EGFR-targeting agents and other anti-cancer therapies. However, the natural and pathological significances of nuclear EGFR in cancers remain mostly unidentified. Efforts to map the relationships at work within the subcellular interactome of EGFR could help us fundamentally understand the mechanisms that govern tumor development and therapeutic resistance, leading to alternative treatment strategies. To address this issue, we employed a label-free spectral counting-based proteomics approach to investigate the EGFR subcellular interactome in a NSCLC cell line. We further examined a selected nuclear EGFR-interacting protein, hnRNP A3, and found that it contributes to the nuclear accumulation of EGFR in NSCLC.

## Results

### Profiling of the subcellular EGFR interactome in CL1-5 cells

**Fig. 1:**
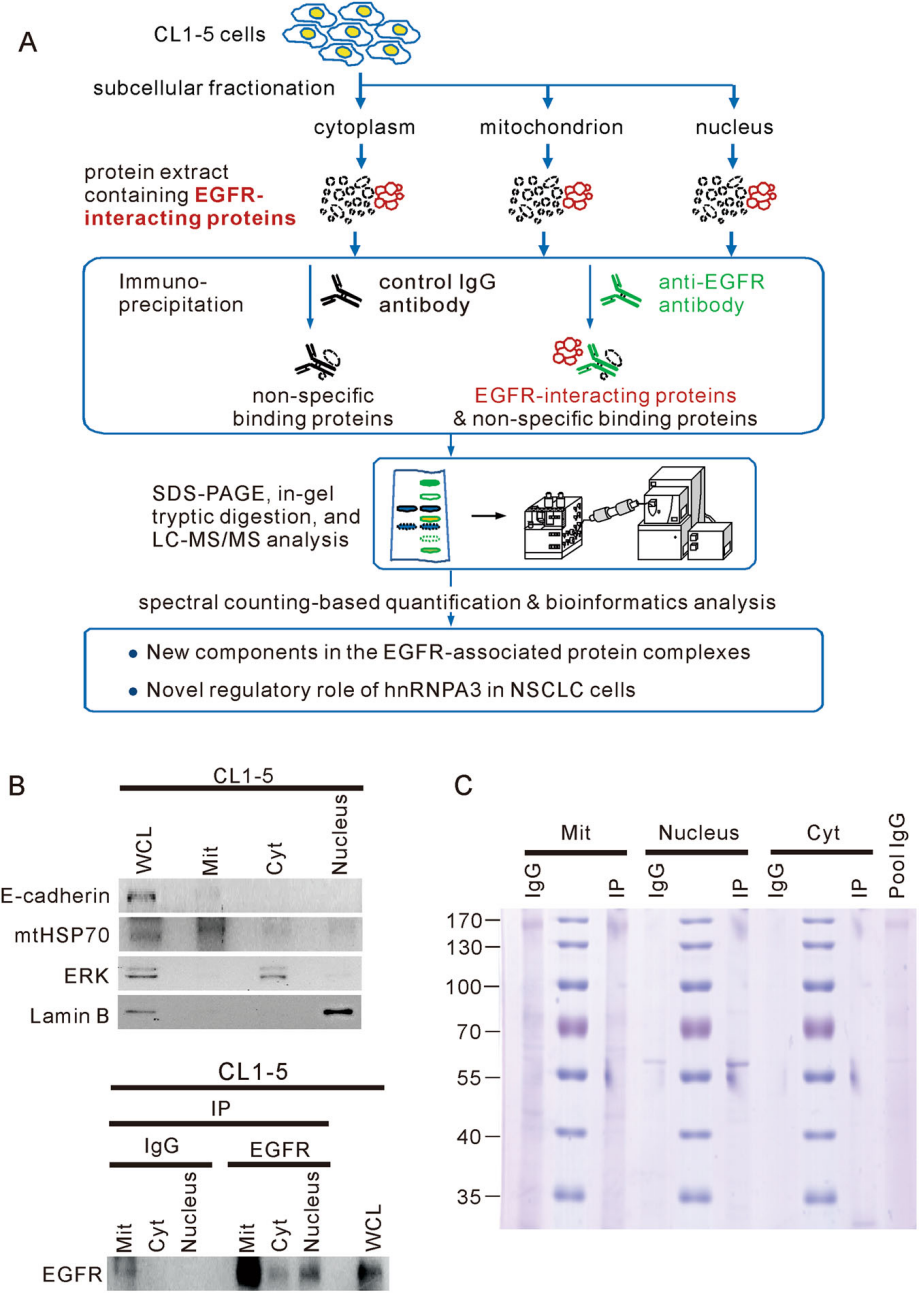
Identification of EGFR-interacting proteins in CL1-5 cells. **a** Illustration of the combined label-free proteomics and experimental approach used to investigate the subcellular interactome of EGFR in NSCLC. Mitochondrial, cytosolic, and nuclear proteins were isolated from CL1-5 cells and immunoprecipitated with anti-EGFR antibody for proteomic analysis. Receptor interactome identification was performed, and specific pairs with high likelihood of interaction were validated experimentally. **b** The proteins from whole cell lysates (WCL), mitochondrial fractions (Mit), cytosolic fractions (Cyt), and nuclear fractions were analyzed for selected markers by Western blotting (top panel). The utilized markers included mtHSP70 for mitochondria, Lamin B for nuclei, E-cadherin for plasma membrane, and ERK for cytoplasm. In the bottom panel, the proteins were immunoprecipitated with anti-EGFR antibody and Western blotting was used to detect EGFR in the immunoprecipitates. **c** The proteins in the immunoprecipitates were separated by SDS-PAGE and stained with Coomassie blue.

The translocation of EGFR to non-canonical subcellular locations, including the nucleus and mitochondria, represents a major yet underappreciated mechanism of NSCLC development. To further understand the putative functions of subcellularly distributed EGFR and EGFR signaling at different subcellular locations, we investigated the EGFR interactome at three subcellular locations (cytosol, mitochondria, and nucleus) to characterize the proteins that interact with the EGFR at these subcellular locations. As previous studies have shown that EGFR are internalized to nucleus and mitochondria without EGF treatment^20–22^, we investigated the subcellular interactome of EGFR in the physiological condition without any stimulation in this study. Figure 1a shows an overview of the strategy used to analyze the EGFR interactome at each subcellular location and functionally explore each identified interacting protein. We used the NSCLC cell line, CL1-5, which is a highly invasive cell line that was derived from CL1-0 cells^23^ and displays higher EGFR expression compared to the less-invasive CL1-0 cell line^24^. Proteins were isolated from the mitochondria, nucleus, and cytoplasm, and immunoprecipitated with an antibody against EGFR (Fig. 1b). The subcellular proteins in the EGFR-immunoprecipitates were separated by sodium dodecyl sulfate-polyacrylamide gel electrophoresis (SDS-PAGE, Fig. 1c), extracted from the gel, and identified by free-labeling approaches combined with liquid chromatography–tandem mass spectrometry (LC–MS/MS).

### Spectral counting-based quantification of identified EGFR-interacting proteins

To further assess the proteins that appeared to interact with EGFR, the relative amounts of proteins identified in the immunoprecipitates were determined by spectral counting-based protein quantification. The fold change for each protein was determined from the ratio of the average spectral count (SC) of the protein in the anti-EGFR fraction versus that in the control IgG fraction. Proteins with fold changes more than two standard deviations (SD) above the mean ratio (i.e., above 6.92, 7.92, and 17.84 for the cytosolic, mitochondrial, and nuclear fractions, respectively) were considered to be EGFR-interacting proteins. Based on the cutoff, 58, 79, and 67 EGFR-interacting proteins were observed in the cytosolic, mitochondrial, and nuclear fractions, respectively (Supplementary Table 1). Among them, 54, 77, and 63 proteins were specific to the cytosolic, mitochondrial, and nuclear groups, respectively (Supplementary Table 1). In contrast, glucose-6-phosphate isomerase was found in the cytosolic and mitochondrial groups; 60S ribosomal protein L6 was detected in the mitochondrial and nuclear groups; and filaggrin, desmocollin-1, and suprabasin were identified in the cytosolic and nuclear groups.

**Table 1:** Biological processes enriched among EGFR-interacting proteins.

Biological process^a^	Identified proteins involved in the process	*p* Value
**Cytoplasm**		
Translation	EGFR, RPL17, RPL27A, RPS15A, RPS9, RPL23A, RPS2, RPS4X, RPS5, RPS3, RPS7, RPS25, EIF3B, RPS19, RPS16, RPS3A, RPS17, RPS14, RPL9, FAU, RPS10, RPS20, RPS23	1.60 × 10^−29^
rRNA processing	RPS19, RPS16, RPS17, RPS14, RPS7	7.90 × 10^−8^
Peptide cross-linking	TGM1, TGM2, DSP, TGM3, FN1	3.42 × 10^−6^
Protein complex biogenesis	JUP, TCP1, RPS14, TUBB2C, TGM2, TGM3, HSPA4, CAT, KPNB1, FLNA	9.26 × 10^−4^
Regulation of apoptosis	EGFR, HMGB1, RPS3A, TUBB2C, CFL1, TGM2, CAT, PRDX1, YWHAE, RPS3	4.78 × 10^−3^
Epidermis development	C1ORF68, FLG, TGM1, DSP, TGM3	6.58 × 10^−3^
**Mitochondria**		
Generation of precursor metabolites and energy	SLC25A12, GPI, NDUFB5, OXA1L, ND4, NDUFV1, SUCLG1, IDH3B, SUCLA2, ATP6V0A2, ETFB, IDH3A	3.83 × 10^−8^
Oxidation reduction	NDUFB5, OXA1L, ALDH18A1, ACADM, ND4, IDH3B, IDH3A, VAT1, COQ6, SLC25A12, ALDH7A1, ALDH1B1, HMOX1, NDUFV1, ACAD9, ETFB	8.59 × 10^−7^
Cofactor metabolic process	DBT, HMOX1, SUCLG1, IDH3B, ACOT1, SUCLA2, IDH3A, COQ6	1.97 × 10^−5^
Mitochondrion organization	OXA1L, OPA1, GFM1, MTX1, BCS1L, DNAJA3	7.80 × 10^−4^
Membrane organization	EGFR, NRCAM, STX4, OXA1L, OPA1, LDLR, MTCH1, SUN2	3.76 × 10^−3^
Transmembrane transport	SLC25A12, CPT2, SLC35B2, SLC25A10, MTCH1, ABCB10, ABCC1, ABCB7, ATP6V0A2	9.66 × 10^−3^
**Nucleus**		
RNA processing/splicing	RALY, TRA2A, SYNCRIP, PNN, PRPF19, SFRS6, NONO, HNRNPA3, HNRNPM, SFRS7, DDX17, DKC1, PRPF8, PCBP1, SFRS9, DHX15, PABPN1, RPL35A, DHX9, EFTUD2, RNPS1, SNW1, CDC5L, SFRS1, HNRNPA0, FBL, PRPF6, RSL1D1, HNRPDL, NOP2, HNRNPUL1, SNRNP200, KHSRP, NOP58, NOP56, PES1, RBM14	2.13 × 10^−36^
Ribonucleoprotein complex biogenesis	RPL35A, GTPBP4, SFRS1, FBL, PRPF6, SFRS6, NOP2, DKC1, SNRNP200, SFRS9, NOP58, NOP56, PES1	8.95 × 10^−12^
Cellular macromolecular complex assembly	SFRS6, H1F0, HP1BP3, SNRNP200, SFRS9, TRIM27, SFRS1, NEFL, PRPF6	3.58 × 10^−4^

^a^The Database for Annotation, Visualization, and Integrated Discovery (DAVID, version 6.7) was applied to functionally annotate enriched proteins, using the annotation category GOTERM_BP_FAT. Processes with at least five protein members and *p* values less than 0.01 were considered significant.

### Bioinformatics analysis of the EGFR interactome

To determine the biological processes that are most likely to be affected by the presence of the EGFR-associated complexes, we used DAVID to annotate the functions of the EGFR-interacting proteins in each subcellular fraction (Supplementary Table 1). The enriched biological processes were as follows: for the cytosolic fraction, translation, rRNA processing, protein complex biogenesis, regulation of apoptosis, and epidermis development; for the mitochondrial fraction, energy generation, oxidation/reduction, mitochondrion organization, membrane organization, and transmembrane transport; and for the nuclear fraction, RNA processing and ribonucleoprotein complex biogenesis (Table 1). Pathway analyses performed using the KEGG database revealed that the EGFR-interacting proteins of the cytosolic fraction were involved in ribosome-related pathways, those of the nuclear fraction were involved in spliceosome-related pathways, and those of the mitochondrial group were related to pathways involved in the metabolism of propanoate, fatty acid, valine, leucine, and isoleucine (Table 2).

**Table 2:** Pathway analysis of the EGFR-interacting proteins.

Term in the KEGG pathway^a^	Identified proteins involved in the pathway	*p* Value
**Cytoplasm**		
Ribosome	RPL17, RPL27A, RPS15A, RPS9, RPL23A, RPS2, RPS4X, RPS5, RPS3, RPS7, RPS25, RPS19, RPS16, RPS3A, RPS17, RPL9, FAU, RPS10, RPS20, RPS23	4.63 × 10^−25^
**Mitochondria**		
Propanoate metabolism	MUT, ALDH7A1, ACADM, ALDH1B1, SUCLG1, SUCLA2	4.55 × 10^−6^
Fatty acid metabolism	ALDH7A1, CPT2, ACADM, ALDH1B1, CPT1A	2.70 × 10^−4^
Valine, leucine and isoleucine degradation	DBT, MUT, ALDH7A1, ACADM, ALDH1B1	3.91 × 10^−4^
**Nucleus**		
Spliceosome	EFTUD2, TRA2A, SNW1, CDC5L, SFRS1, PRPF6, HNRNPA3, SFRS6, PRPF19, SFRS7, HNRNPM, PCBP1, PRPF8, SNRNP200, SFRS9, DHX15, ACIN1	5.01 × 10^−22^

^a^DAVID was applied to functionally annotate the enriched proteins. The knowledge base used was the KEGG pathway database. Processes with at least five protein members and *p* values less than 0.01 were considered significant.

**Fig. 2:**
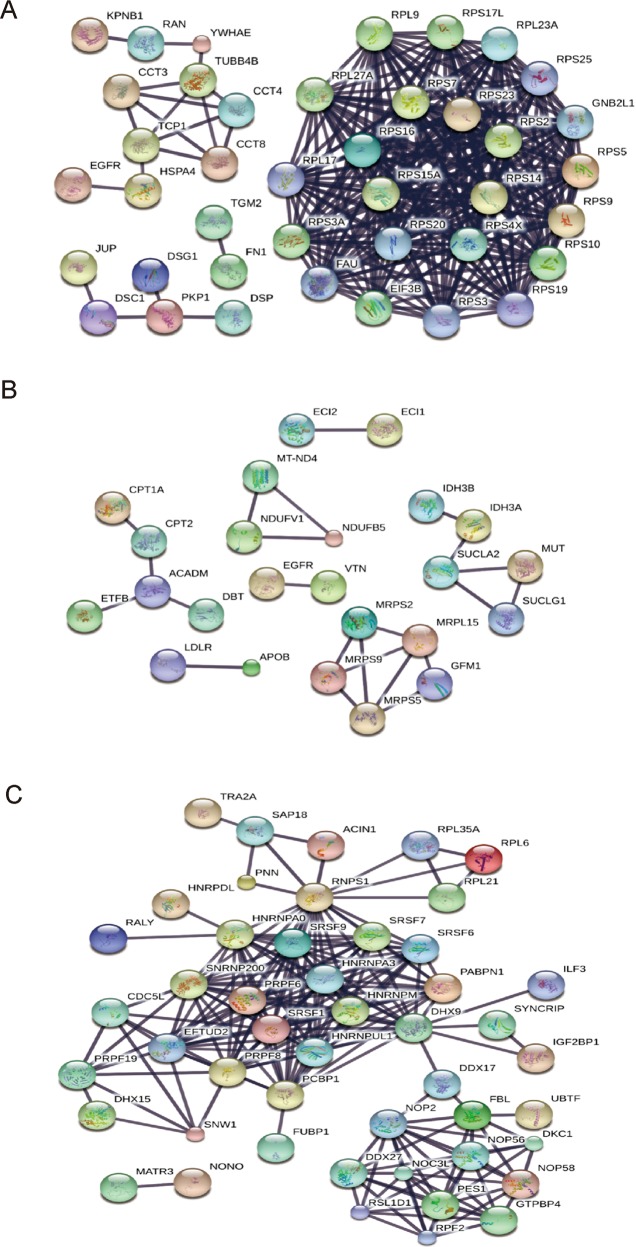
Protein–protein interaction (PPI) network analysis of EGFR-interacting proteins. PPI networks of the EGFR-interacting proteins identified in the cytoplasm (**a**), mitochondria (**b**), and nucleus (**c**) were constructed using the STRING v10.5 database. A combined score >0.9 (indicating the highest confidence) was used as the cutoff criterion. The interaction links between individual nodes/proteins are shown as solid lines.

We further used the STRING online database to establish a network of protein–protein interactions (PPIs) between the identified EGFR-interacting proteins (Supplementary Table 1). The analyses yielded 268, 24, and 204 strong interaction links between the EGFR-interacting proteins identified in the cytosolic, mitochondrial, and nuclear fractions, respectively (Fig. 2). In line with the results from our DAVID and KEGG analyses (Tables 1, 2), the STRING analysis generated a module that depicted interactions between EGFR and proteins grouped into the RNA processing/splicing and ribonucleoprotein complex biogenesis interaction networks (Fig. 2). The RNA processing/splicing group primarily included hnRNP family proteins, such as hnRNP A0, hnRNP A3, hnRNP DL, hnRNP M, and hnRNP UL1. The highest score was found for hnRNP A3, which is involved in RNA processing/splicing and the spliceosome (Tables 1, 2). Since hnRNP A3 is reportedly overexpressed in lung cancer^25^ and has been shown to interact with nuclear EGFR in A549 cells^19^, we selected hnRNP A3 for further study, and set out to examine the functional role of its interaction with nuclear EGFR.

### Expression levels of hnRNP A3 and EGFR in paired NSCLC tumor and adjacent normal tissues

**Fig. 3:**
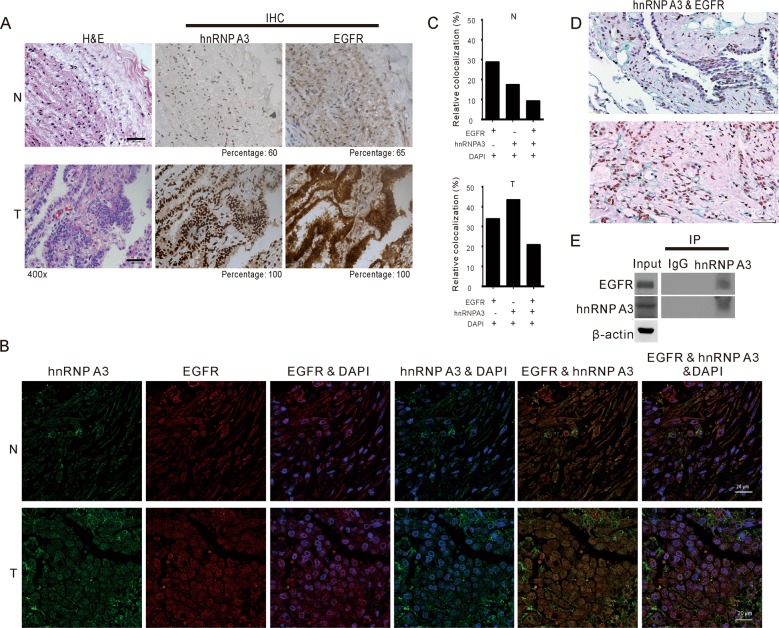
Immunohistochemical (IHC) and immunofluorescence (IF) staining of EGFR and hnRNP A3 in NSCLC tumor and adjacent normal tissues. **a** Tumor (T) and adjacent normal (N) sections from an overall stage 1 patient were examined by hematoxylin & eosin staining (H&E) and IHC staining (magnification, ×400) for the detection of hnRNP A3 and EGFR. The immunoreactivity of hnRNP A3 and EGFR in tumor (T) and adjacent normal (N) of IHC staining was scored and indicated in each panel. **b** IF staining was used to assess the expression levels of hnRNP A3 and EGFR from an overall stage 3 patient. **c** The colocalization of hnRNP A3, EGFR, and 4′,6-diamidino-2-phenylindole (DAPI) from (**b**) was analyzed using the MetaMorph software. **d** IHC double staining was used to detect the colocalization of hnRNP A3 and EGFR from the tumor (bottom) and the hyperplasia (top) sections of a NSCLC patient. The green color represents EGFR signal and the brown color represents hnRNP A3 signal. The deep-blue color (green plus brown) indicates the colocalization and are marked with arrows. Magnification: ×400. **e** Whole cell lysates were prepared from a frozen NSCLC tissue and immunoprecipitated (IP) with anti-hnRNP A3 or anti-IgG as control. The immuno-precipitated proteins were then analyzed by Western blot.

To address the functional role of the putative EGFR–hnRNP A3 interaction in the nucleus, we used immunohistochemistry (IHC) to detect the expression levels of hnRNP A3 and EGFR in 15 NSCLC tumor tissues and paired adjacent normal sections. The clinical characteristics of the patients are summarized in Supplementary Table 2. Representative IHC results for hnRNP A3 and EGFR (brown staining) from an overall stage 1 patient are shown in Fig. 3a. The percentage of positive staining ranged from 0 to 100% in all samples. The clinical relevance of hnRNP A3 and EGFR expression in paired NSCLC tumor and adjacent normal tissue samples is summarized in Supplementary Table 3. Elevated expression of hnRNP A3 and EGFR was detected in the tumor section compared with the adjacent normal section. To determine if hnRNP A3 showed nuclear colocalization with EGFR in NSCLC, we used immunofluorescence (IF) staining to examine the expression patterns of these proteins in the paired tumor and adjacent normal tissues of an overall stage 3 patient. As shown in Fig. 3b, c, hnRNP A3 and EGFR showed elevated colocalization in tumor sections compared with adjacent normal sections. The elevated colocalization of hnRNP A3 and EGFR in tumor sections was also examined by IHC double staining in a NSCLC patient. As shown in Fig. 3d, the colocalization of EGFR and hnRNP A3 was clearly much higher in the tumor sections than the hyperplasia sections. In addition, the tissue extract was prepared from a frozen NSCLC tissue and immunoprecipitated with anti-hnRNP A3 or anti-IgG. As shown in Fig. 3e, EGFR was readily detected in the immunoprecipitates pulled down by anti-hnRNP A3. These results suggest that nuclear hnRNP A3 and EGFR interact in NSCLC.

### hnRNP A3 and EGFR interact in the nuclei of NSCLC cells

**Fig. 4:**
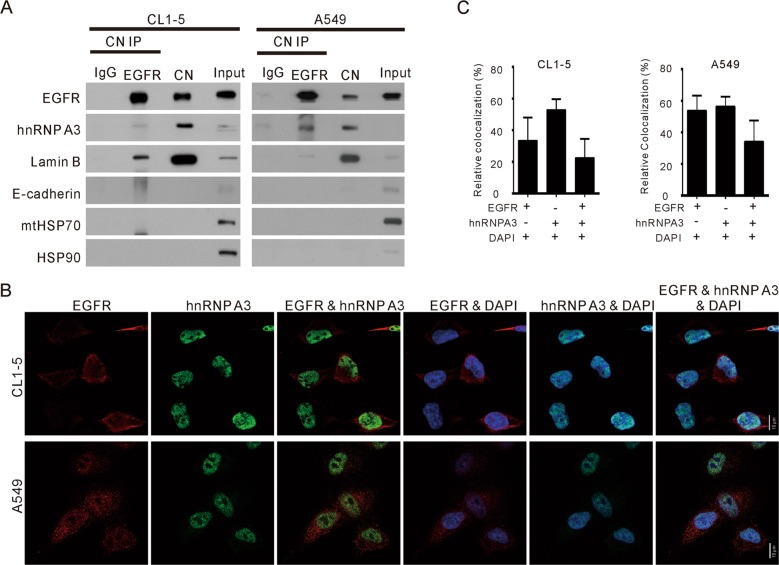
hnRNP A3 interacts with EGFR in the nucleus. **a** Whole cell lysates from CL1-5 and A549 cells were further processed to obtain nuclear fractions. The proteins from each nuclear fraction (CN; 1 mg) were immunoprecipitated (IP) with anti-EGFR or anti-IgG (control), and the proteins in the immunoprecipitates were analyzed by Western blotting. The tested markers included Lamin B for nuclei, E-cadherin for plasma membrane, mtHSP70 for mitochondria, and HSP90 for cytoplasm. **b** IF staining were was used to assess the subcellular distributions of hnRNP A3, EGFR, and DAPI (nuclei) in CL1-5 and A549 cells. Colocalization of hnRNP A3 and EGFR in the DAPI nucleus is seen as a white-colored spot, and **c** the colocalization of hnRNP A3, EGFR, and DAPI in ~100 cells was analyzed using the MetaMorph software.

To confirm that hnRNP A3 interacts with EGFR in the nucleus, we immunoprecipitated the nuclear proteins of CL1-5 and A549 cells using an antibody against EGFR. As shown in Fig. 4a, hnRNP A3 was detected in EGFR immunoprecipitates from both CL1-5 and A549 cells. To further evaluate the nuclear colocalization of EGFR with hnRNP A3, we used *in situ* IF staining to analyze the subcellular distributions of EGFR, hnRNP A3 and DAPI (a nuclear marker). As shown in Fig. 4b, hnRNP A3 and EGFR were highly colocalized in the nuclei of both cell lines. As shown in Fig. 4c, about 35 and 55% of EGFR were localized in the nucleus of CL1-5 and A549 cells, respectively. Three-channel colocalization analysis indicated that about 20 and 30% of colocalization of EGFR, hnRNP A3, and DAPI in CL1-5 and A549 cells, respectively. Taken together, these results, together with those from our analyses of the interactome and clinical tissues, strongly indicate that hnRNP A3 and EGFR interact in vitro and in vivo.

### hnRNP A3 is essential for the nuclear translocation of EGFR

**Fig. 5:**
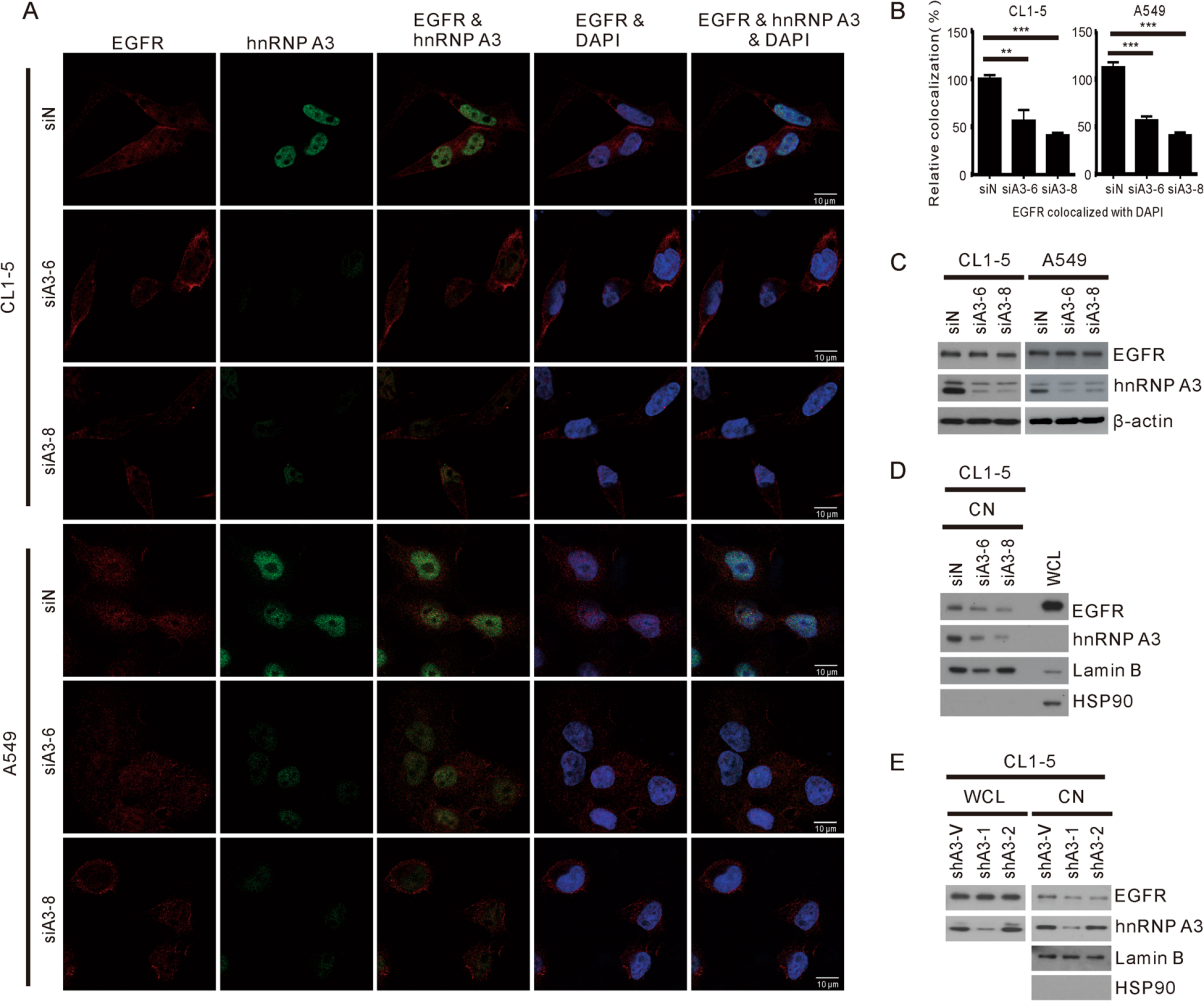
Effects of hnRNP A3 downregulation on translocation of EGFR into the nucleus. CL1-5 and A549 cells were transfected with si-hnRNP A3 (siA3-6 and siA3-8) or siN (control). At 72 h post-transfection, the cells were IF stained with anti-EGFR and -hnRNP A3 antibodies. Representative IF staining results are shown in (**a**). The scores for the localization of EGFR in nucleus from ~100 cells were calculated using the MetaMorph software. The summary data shown in (**b**) indicate the means ± SD from three independent experiments; **p < 0.01 and ***p < 0.001, as assessed with the Student’s *t*-test. **c** Western blot analysis for the expression level of hnRNP A3 and EGFR. β-Actin was used as a loading control. **d** The lysates of CL1-5 cells transfected with si-hnRNP A3 were fractionated to isolate nuclear fraction for Western blot analysis. **e** CL1-5 cells were infected with sh-hnRNP A3 (sh-A3-1 and sh-A3-2) or sh-V (control) and stable clones of hnRNP A3-knockdown cells were obtained. The levels of relevant proteins in nuclear fractions (CN, 10 μg) and WCL (20 μg) were analyzed by Western blot analysis. The cytoplasmic marker, HSP90, and nuclear marker, Lamin B, were included to validate the purity of the nuclear fraction.

As hnRNP A3 has been shown to shuttle cargo between the cytosol and nucleus^26–28^, we postulated that it might perform this function for EGFR. To test this hypothesis, we used IF staining to examine the effects of hnRNP A3 knockdown on the cellular distribution of EGFR. As shown in Fig. 5a, b, the colocalization of EGFR with DAPI was decreased in CL1-5 and A549 cells depleted of hnRNP A3. To confirm these IF staining results, we employed nuclear fractionation and Western blot analysis to examine the expression levels of EGFR and hnRNP A3 in nuclear and whole-cell lysates (WCL). As shown in Fig. 5c, while the level of hnRNP A3 was greatly reduced in the hnRNP A3-depleted cells, the level of EGFR in the whole-cell lysates remained unchanged. In contrast, the levels of nuclear EGFR were greatly reduced in hnRNP A3-depleted CL1-5 cells compared to the siN control (Fig. 5d). As the total level of EGFR was not affected by the depletion of hnRNP A3, it is likely that the reduced localization of EGFR in the nucleus of hnRNP A3-depleted cells was accompanied by the redistribution of EGFR to membrane, cytoplasm, or cytoplasmic organelles. In addition, we also examined how stable depletion of hnRNP A3 affected the nuclear localization of EGFR. CL1-5 cells stably depleted of hnRNP A3 by sh-hnRNP A3 (shA3-1 and shA3-2) were subjected to nuclear fractionation, and the levels of EGFR in nuclear and whole-cell lysates were assayed by Western blot analysis. As shown in Fig. 5e, the levels of total EGFR in WCL of shA3-1- and shA3-2-depleted CL1-5 cells were similar to that of the sh-V control, whereas the levels of nuclear EGFR were greatly reduced in shA3-1- and shA3-2-depleted CL1-5 cells compared to the sh-V control. Collectively, these results show that hnRNP A3 modulates the nuclear localization of EGFR in NSCLC.

### Effects of hnRNP A3 depletion on the cell proliferation, anchorage-independent growth, and in vivo tumor growth of NSCLC

**Fig. 6:**
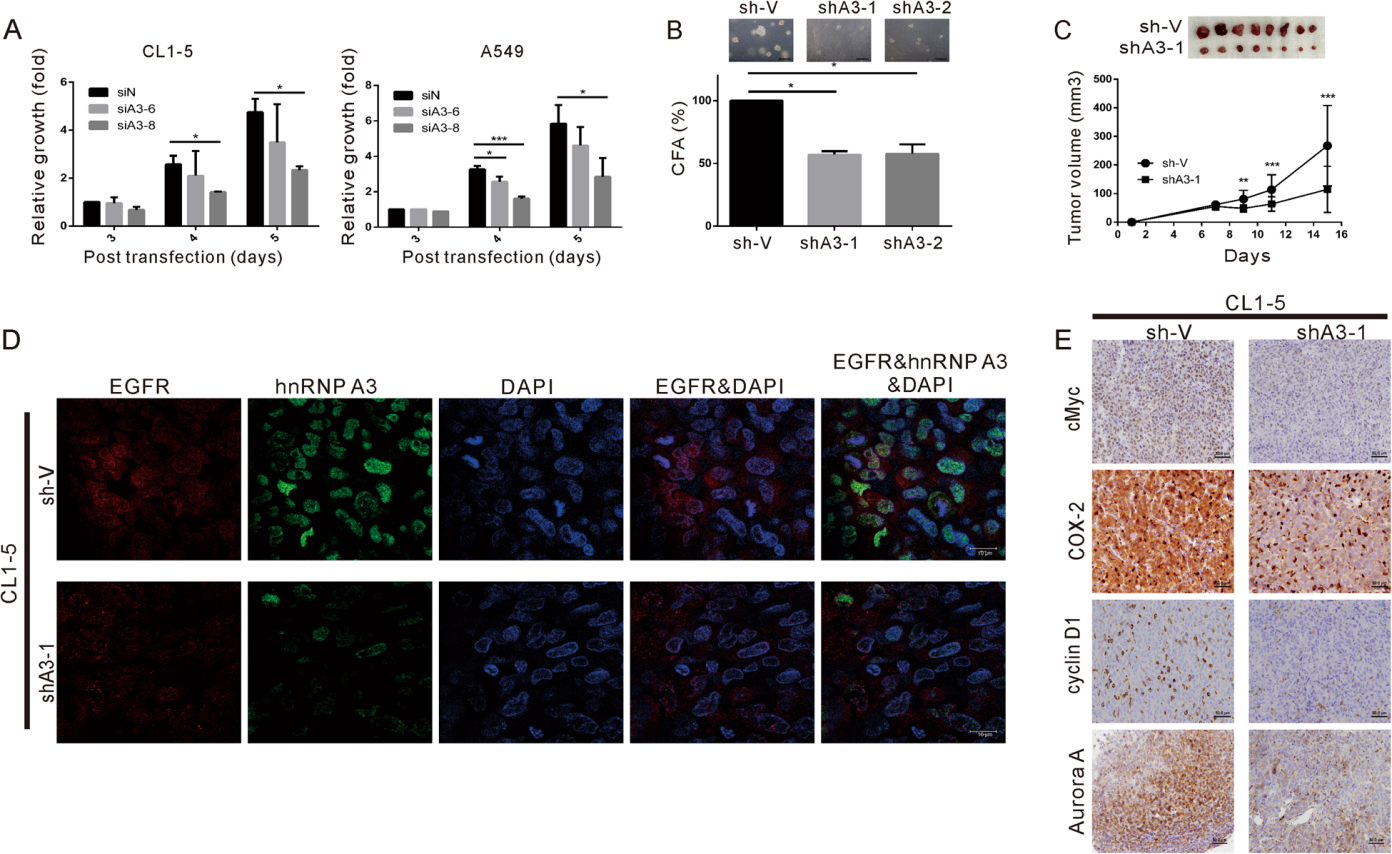
Effect of hnRNP A3 downregulation on cell proliferation and tumorigenesis in vitro and in vivo. **a** CL1-5 and A549 cells were transfected with si-hnRNP A3 (siA3-6 and siA3-8) or siN (control). After being cultured for 48 h, the transfected cells were monitored for cell proliferation. **b**, **c** CL1-5 cells were infected with sh-hnRNP A3 (shA3-1 and shA3-2) or empty vector (sh-V) and stable clones of hnRNP A3-knockdown cells were obtained. The ability of these cells to perform anchorage-independent growth ability in soft agar was examined, as shown in (**b**). The growth of these cells in subcutaneously implanted mice is shown in (**c**). **d** IF staining of EGFR, hnRNP A3, and DAPI in excised xenograft tissues. **e** IHC staining of cyclin D1, COX-2, aurora A, and c-Myc in excised xenograft tissues. Magnification: ×400. The data shown in (**a**–**c**) represent the means ± SD from three independent experiments; *p < 0.05, **p < 0.01, and ***p < 0.001 as assessed using the Student’s *t*-test.

As hnRNP A3 is reportedly overexpressed in lung cancer^25^, we next examined its effects on cell proliferation and anchorage-independent growth in CL1-5 and A549 cells. As shown in Fig. 6a, transient depletion of hnRNP A3 inhibited cell proliferation in CL1-5 cells (Fig. 6a, left panel) and A549 cells (Fig. 6a, right panel). Similarly, stable depletion of hnRNP A3 by shRNA suppressed the anchorage-independent growth ability of CL1-5 cells (Fig. 6b). These results suggest that hnRNP A3 may be involved in the tumorigenesis of NSCLC. To determine if hnRNP A3 affects NSCLC tumorigenesis in vivo, we examined how stable depletion of hnRNP A3 affected tumor growth in a xenograft mouse model. As shown in Fig. 6c, the growth of shA3-1 cell-derived tumors was slower than that of sh-V control cell-derived tumors. Similarly, the excised shA3-1 tumors were considerably smaller than the sh-V control tumors. To evaluate if the depletion of hnRNP A3 also reduced the nuclear localization of EGFR, we subjected the excised tumors to IF staining of hnRNP A3 and EGFR. As shown in Fig. 6d, e, the levels of hnRNP A3, nuclear EGFR, cMyc, cyclin D1, aurora A, and COX-2 were greatly decreased in shA3-1 tumors compared to sh-V tumors. These results indicate that the downregulation of hnRNP A3 reduced tumor growth in vivo, possibly by decreasing the levels of nuclear EGFR and its target regulation, including cMyc, cyclin D1, aurora A, and COX-2.

## Discussion

In this study, we examined the EGFR interactome at three subcellular locations (cytosol, mitochondria, and nucleus) in order to deduce the functionality of EGFR at these subcellular locations. Using free-labeling approaches combined with LC–MS/MS, we identified 58, 79, and 67 EGFR-interacting proteins in the cytosol, mitochondria, and nucleus, respectively (Supplementary Table 1). Our enrichment analysis of categories belonging to biological processes revealed that the cytosolic EGFR-interacting proteins were associated with translation, rRNA processing, peptide cross-linking, protein complex biogenesis, regulation of apoptosis, and epidermis development (Table 1). Pathway analyses using the KEGG database revealed that the cytosolic EGFR-interacting proteins were involved in ribosome-related pathways (Table 2). Our findings suggested a previously unknown function of EGFR, namely that cytosolic EGFR may interact with ribosomal proteins to promote the translational program in NSCLC. In tumor cells, the activation of survival signaling pathways increases overall protein synthesis and enhances cellular metabolism, tumor growth, and metastasis^29–31^, and the deregulation of translation can enable tumors to resist clinical treatment^32^. In this regard, it is interesting to speculate that the deregulation of translation and ribosomes may contribute to drug resistance, especially in the NSCLC cells harboring constitutively activating mutations of EGFR.

The EGFR-interacting proteins in mitochondria were found to correlate with precursor metabolite generation, energy production, and mitochondrion organization (Table 1). The mitochondrial EGFR-interactome included Dnaja3 (Table 1, also known as Tid-1); this protein has been shown to govern the mitochondrial localization of EGFR, and the mitochondrial accumulation of EGFR has been shown to promote metastasis in NSCLC^33^. While the functions of mitochondrial EGFR remain poorly understood, our pathway analyses revealed that many of the mitochondrial EGFR-interacting proteins are involved in the metabolism of propanoate, fatty acids, and degradation of valine, leucine, and isoleucine (Table 2). The palmitoylation of mitochondrial EGFR has been shown to induce mitochondrial fusion and promote cell survival in prostate and breast cancer^34^. The products of valine, leucine, and isoleucine catabolism may enter the citric acid cycle and link to the metabolism of propanoate and fatty acids^35^. In the future, it could be interesting to investigate exactly how mitochondrial EGFR modulates these metabolic pathways to promote metastasis.

The EGFR-interacting proteins in the nucleus were found to be involved in RNA processing/splicing, ribonucleoprotein complex biogenesis, and cellular macromolecular complex assembly (Table 1). Pathway analyses revealed that the nuclear EGFR-interacting proteins were involved in spliceosome-related pathways (Table 2). Consistent with this finding, our STRING-based EGFR interactome profiling generated an EGFR interaction module that grouped into the RNA processing/splicing and ribonucleoprotein complex biogenesis interaction networks (Fig. 2). The RNA processing/splicing group primarily included hnRNP family proteins, including hnRNP A0, hnRNP A3, hnRNP DL, hnRNP M, and hnRNP UL1. Several members of hnRNP A/Bs, which are comprised of A1, A2, A3, and A0^36^ have been identified in the nuclear EGFR interactome (Supplementary Table 1). HnRNP A1, A2, and A3 have been shown to copurify with the splicing complexes^37,38^. The functions of hnRNP A1 and A2 in the splicing of oncogenes and tumor-related genes may explain the frequent dysregulation of hnRNP A/Bs in different types of cancers^39,40^. HnRNP A3 has been reported to interact with nuclear EGFR and stabilize the mRNA involved in the aerobic glycolysis in response to irradiation^19^. However, compared to the well-studied members of hnRNP A1 and A2, the role of hnRNP A0 and A3 in RNA processing/splicing are poorly understood. In this study, hnRNP A3 was detected as a spliceosome component that interacted with nuclear EGFR and is predicted to function in a related manner similar to that of hnRNP A1 and A2. Nuclear EGFR has also been shown to regulate the stability of mRNAs related to the VEGF pathway in stress-exposed NSCLC and head and neck cancer cell lines^41^. It is also quite possible that the ability of nuclear EGFR to activate the expression of cyclin D1^9^, inducible nitric oxide synthase^2^, B-Myb^11^, COX-2^15^, aurora A^12^, c-Myc^16^, and breast cancer resistance protein^17^ may be related to this RNA processing function.

From among the nuclear EGFR-interacting proteins, we selected hnRNP A3 for further study. Our results suggest that hnRNP A3 is involved in the nuclear accumulation of EGFR in NSCLC. For example, IF staining of EGFR and hnRNP A3 in tumor and adjacent normal tissues obtained from NSCLC patients revealed that hnRNP A3 and EGFR exhibited elevated colocalization in tumor sections compared with adjacent normal sections (Fig. 3). Consistent with a previous study, hnRNP A3 was detected predominantly in nucleus with a minor expression in the cytosol^42^. Depletion of hnRNP A3 did not affect the total level of EGFR, but reduced the nuclear accumulation of EGFR (Fig. 5). These data suggest that the reduced nuclear EGFR accumulation is accompanied with increased relocation of EGFR to membrane, cytoplasm, or cytoplasmic organelles. Depletion of hnRNP A3 also reduced anchorage-independent growth ability and tumor cell growth both in vitro and in vivo (Fig. 6). These results suggest that nuclear EGFR plays an important role in the tumorigenesis of NSCLC. Since hnRNP A/Bs have been reported to be overexpressed in lung cancer^43^, our identification of hnRNP A/Bs as nuclear EGFR interacting proteins suggests that such an interaction may involve yet-to-be identified mechanism to facilitate the functions of hnRNP A/Bs in mRNA processing/trafficking. Nuclear EGFR may employ its kinase activity to phosphorylate hnRNP A/Bs or it may function as transcriptional regulator to regulate the expression of hnRNP A/Bs. Thus, the phosphorylation status and the expression levels of hnRNP A/Bs may be affected by the nuclear EGFR.

In summary, we herein examined subcellular EGFR interactomes, analyzed the putative functions of EGFR at these subcellular locations, and report that a nuclear EGFR-interacting protein selected for further study, hnRNP A3, modulates nuclear EGFR accumulation and tumor growth in NSCLC.

## Materials and methods

### Cell lines and culture media

The human NSCLC cell line, CL1-5, was established from CL1-0 cells via selecting for increased invasive capability using a Transwell chamber assay^23^. These cells were kindly provided by Dr. Pan-Chyr Yang (Department of Internal Medicine, College of Medicine, National Taiwan University, Taipei, Taiwan). A549 cells were obtained from the American Type Culture Collection (Manassas, VA, USA). CL1-5 and A549 cells express wild-type EGFR^44^. In the absence of ligand stimulation, EGFR was not phosphorylated in these cells. CL1-5 cells were cultivated in RPMI-1640 supplemented with 10% fetal bovine serum (FBS) and 100 U/ml penicillin and streptomycin together. A549 cells were cultured in Dulbecco’s modified Eagle’s medium containing 10% FBS, 2.5 mM l-glutamine, 0.5 mM sodium pyruvate, and 100 U/ml penicillin and streptomycin. All cell lines were confirmed by short tandem repeat analysis and mycoplasma PCR. All culture media and FBS were purchased from Life Technologies (Grand Island, NY, USA).

### Antibodies

Antibodies against EGFR (1005), lamin B (C-20), hnRNP A3 (A15), ERK (K-23), E-cadherin, c-Myc (N-262), cyclin D1 (HD11), and HSP90 were purchased from Santa Cruz Biotechnology, Inc. (Santa Cruz, CA, USA). The antibody against mitochondrial HSP70 (MA3-02) was purchased from Thermo Fisher Scientific, Inc. (Waltham, MA, USA). The antibody against COX-2 (ab15191) and aurora A (35C1) were purchased from Abcam (Cambridge, UK). The antibody against β-actin was purchased from Sigma (St. Louis, MO, USA). Alexa Fluor® 594 donkey anti-rabbit IgG (A21207), Alexa Fluor® 647 donkey anti-goat IgG (A21447), and Alexa Fluor® 488 goat anti-mouse IgG (A11001) were purchased from Life Technologies-Molecular Probes.

### Tumor specimens

The 15 paired tumor and adjacent normal tissues were obtained from NSCLC patients who underwent surgical resection at Chang Gung Memorial Hospital. This study was approved by the Ethics Committee of Chang Gung Memorial Hospital. Written informed consent was obtained from all patients.

### Subcellular fractionation

Nuclear, cytoplasmic, and mitochondrial fractions were obtained from lung cancer cells using a Qproteome mitochondria isolation kit (Qiagen, Venlo, Netherlands) according to the manufacturer’s protocol^33^. Cells were suspended in a lysis buffer, which selectively disrupts the cell membrane without solubilizing it, resulting in the isolation of cytosolic proteins.

### Western blotting, immunoprecipitation, and immunofluorescence staining

Western blotting, immunoprecipitation, and immunofluorescence (IF) were performed as described previously^24,33^. The distributions of hnRNP A3 and EGFR in NSCLC tissues and cells were determined by IF staining as described previously^33^. Cells or tissues were visualized under confocal microscopy (LSM 700; Carl Zeiss, Jena, Germany) and the MetaMorph software was used to examine colocalization (MetaMorph Inc., Nashville, TN, USA). The analysis colocalization of EGFR (red) and hnRNP A3 (green) in nucleus (DAPI/blue) using Metamorph software are illustrated in Supplementary Fig. S1.

### Immunohistochemical assay

Immunohistochemical staining for EGFR and hnRNP A3 was performed as described previously^24^. These tissues were examined for the extent of EGFR and hnRNP A3 staining by a pathologist (W. Y. Chuang) in a blinded manner^45^. The immunoreactivity for the hnRNP A3 and EGFR was semiquantitatively scored by the percentage of positive-staining tumor cells in a representative large section of each tissue specimen. The immunoreactivity was grouped into four groups according to the percentage of the positive tumor cells: negative (0%), low (1–50%), medium (51–95%), and high (96–100%). IHC double staining was performed with MultiView (mouse-HRP/rabbit-DAB) IHC kit (Enzo Life Sciences, Inc., NY, USA), by following the protocols provided by the manufacturer.

### In-gel digestion and mass spectrometric analysis

The immunoprecipitates were separated by 10% SDS-PAGE and stained with a Colloidal Blue Staining Kit (Thermo Fisher Scientific). Destaining was performed with 10% methanol and 5% acetic acid, each gel lane was cut into 10 pieces, and each piece was further separated into two replicates. The pieces were then dehydrated in acetonitrile (Mallinckrodt Baker) and dried in a SpeedVac. Proteins were reduced with 25 mM NH_4_HCO_3_ containing 10 mM dithiothreitol at 60 °C for 30 min, alkylated with 55 mM iodoacetamide at room temperature for 30 min, and then digested by trypsin (20 μg/ml; Promega, Madison, WI, USA) overnight at 37 °C. The digested peptides were extracted by acetonitrile and dried in a SpeedVac.

The extracted peptides were identified by LTQ-Orbitrap Discovery (Thermo Fisher, Waltham, MA) coupled with high-performance liquid chromatography. Briefly, peptide extracts were reconstituted in solution A (0.1% formic acid), loaded across a trap column (Zorbax 300SB-C_18_, 0.3 × 5 mm^2^; Agilent Technologies, Taiwan) at a flow rate of 0.2 μL/min in solution A, and separated on a resolving 100-mm analytical C_18_ column (inner diameter, 75 μm) using a 15-μm tip (New Objective, Woburn, MA, USA). The peptides were eluted with a 60-min gradient at a flow rate of 0.25 μL/min. The LTQ Orbitrap was operated using the Xcalibur 2.0 software (Thermo Fisher). The data were acquired in a data-dependent mode containing one MS scan, using the Orbitrap at a resolution of 30,000 and 10 MS/MS scans (in the linear ion trap) for the 10 most abundant precursor ions. The *m*/*z* scan range for the MS scans was set as 350–2000 Da, and the ion signal of (Si(CH_3_)_2_O)_6_H^+^ at *m*/*z* 445.120025 was used as a lock mass for internal calibration. To increase identification coverage, the precursor ions selected for MS/MS analysis were dynamically excluded for 180 s^46–48^.

### Database searching and protein identification

The obtained spectra were searched with the Mascot algorithm (Version 2.1; Matrix Science, Boston, MA, USA) against the Swiss-Prot human sequence database (released March 2018, selected for *Homo sapiens*, 20,198 entries) of the European Bioinformatics Institute. The peak list was generated using the Thermo ExtractMSn software (Version 1.0.0.8, May 2012 release). The mass tolerances for parent and fragment ions were set as 10 ppm and 0.5 Da, respectively. Oxidation on methionine (+15.99 Da) and carbamidomethylation on cysteine (+57 Da) were set as variable and fixed modifications, respectively. The enzyme was set as trypsin and up to one missed cleavage was allowed. The random sequence database was used to estimate false positive rates for protein matches. The resulting files were further integrated using the Scaffold software (Version 4.2.1; Proteome Software, Portland, OR, USA), which included the PeptideProphet algorithm^49^ for assignment of peptide MS spectra and the ProteinProphet algorithm^50^ for grouping peptides to a unique protein or protein family (if the peptides are shared among several isoforms). The thresholds for PeptideProphet and ProteinProphet probability were set as 0.95 to ensure an overall false discovery rate below 0.5%. Only proteins with two unique matching peptides were retained.

### Spectral counting-based protein quantification and bioinformatics analysis

To identify the binding partners of EGFR, we compared the protein levels between immunoprecipitation products of the control vector and EGFR vector groups using a previously described label-free spectral counting-based quantification method^51^. Briefly, the exclusive spectrum count for each identified protein was exported from the Scaffold software in Excel format (Microsoft, Redmond, WA, USA). To reduce differences between analyses, the normalized spectral count (NSC) of each protein was calculated by the spectral count (SC) for that protein divided by the total SC of the analysis. The fold change was estimated as the ratio of the average of normalized SCs in the EGFR group to that in the control group. Because not all proteins were identified in all replicates, the SCs of unidentified proteins or missing values in a certain sample were assigned a value of one; this enabled us to avoid overestimating the fold changes and dividing by zero.

The biological pathways and processes involved with the EGFR-interacting proteins were revealed using the Kyoto Encyclopedia of Genes and Genomes (KEGG) database (http://www.genome.jp/kegg/pathway.html) and the Database for Annotation, Visualization, and Integrated Discovery (DAVID, version 6.8, https://david.ncifcrf.gov/), respectively^52^. The known and predicted associations between the EGFR-interacting partners were analyzed with the STRING online software (version 10.5, https://string-db.org/cgi/input.pl). A combined confidence score of ≥0.9 was used as the cutoff criterion^53^.

### RNA interference (RNAi)

hnRNP A3 was downregulated using RNAi from Dharmacon (Lafayette, CO, USA), with a nontargeting siRNA (D-001810-01, Dharmacon) used as a control. The two siRNAs targeting the human hnRNP A3 mRNA covered nucleotides 785–803 from the start codon (A3-6: GGAGGGAACUUUGGAGGUG) and nucleotides 1041–1059 (A3-8: ACAAUGAAGGAGGAAAUUU). Transfection was performed by using the Dharmafect 1 transfection reagent (Dharmacon) according to the manufacturer’s instructions, as described previously^54^.

### Plasmids and establishment of stable knockdown subclones

Stable knockdown of hnRNP A3 was achieved using a short hairpin RNA approach (shRNAs). Packed lentiviruses expressing shRNAs designed to knock down hnRNP A3 (TRCN0000245295 and TRCN0000074511) were obtained from the RNA interference consortium (National RNAi Core Facility, Academia Sinica, Taipei, Taiwan). Cells were infected with lentivirus and cultured in the presence of puromycin for 2 weeks. The stable knockdown CL1-5 cells were designated shA3-1 and shA3-2, while the control was called sh-V (control).

### Assays for cell viability and anchorage-independent growth

Trypan blue exclusion assay for viable cell counts and soft agar colony formation assays for anchorage-independent growth were performed as described previously^55,56^.

### Subcutaneous xenografts

Each xenograft was established by subcutaneously injecting 2 × 10^6^ cells into the right flank of 6-week-old male Balb/c nude mice (*n* = 8 per group, National Laboratory Animal Center, Taipei, Taiwan). Tumor volumes were measured twice a week using calipers, and tumor volumes were calculated according to the formula *V* = 0.5 × *W*^2^*L*, where *W* was taken as the smaller diameter and *L* was taken as the larger diameter. Mice were sacrificed by CO_2_ asphyxiation on day 15 after inoculation. All animal experiments were performed according to the guidelines for the Animal Care Ethics Commission of Chang Gung Memorial Hospital.

### Statistical analysis

The presented results represent three independent experiments with similar results. Statistical differences were evaluated using the Student’s *t*-test and considered significant at *p* < 0.05.

## Supplementary information

Supplementary Table 1, 2, and 3

Supplementary Figure 1
