# Supplementary material for: Profiling of subcellular EGFR interactome reveals hnRNP A3 modulates nuclear EGFR localization
Source: Oncogenesis. 2020 Apr 22;9(4):40. doi: 10.1038/s41389-020-0225-0 (PMC7176650; doi:10.1038/s41389-020-0225-0)
Supplement: Supplementary file 1 — Supplementary Table 1, 2, and 3 [file 41389_2020_225_MOESM1_ESM.docx]

| **Supplementary Table 1. List of EGFR-interacting proteins** | | | | | | | | |
| --- | --- | --- | --- | --- | --- | --- | --- | --- |
| Accession No. | Protein name | Gene name | M.W.  (kDa) | Protein  probability | No. of  unique  peptides | No. of  unique  spectra | No. of  total  spectra | Sequence  coverage |
| Cytoplasmic fraction | | | | | | | | |
| TGM2_HUMAN | Protein-glutamine gamma-glutamyltransferase 2 | TGM2 | 77.3 | 100.0% | 6 | 6 | 13 | 11.50% |
| DSC1_HUMAN | Desmocollin-1 | DSC1 | 100.0 | 100.0% | 6 | 6 | 10 | 7.27% |
| SBSN_HUMAN | Suprabasin | SBSN | 60.5 | 100.0% | 2 | 2 | 8 | 6.10% |
| RS14_HUMAN | 40S ribosomal protein S14 | RPS14 | 16.3 | 100.0% | 4 | 4 | 8 | 19.20% |
| GNA11_HUMAN | Guanine nucleotide-binding protein subunit alpha-11 | GNA11 | 42.1 | 100.0% | 2 | 2 | 6 | 5.29% |
| RS10_HUMAN | 40S ribosomal protein S10 | RPS10 | 18.9 | 100.0% | 4 | 5 | 6 | 27.90% |
| PKP1_HUMAN | Plakophilin-1 | PKP1 | 82.9 | 100.0% | 3 | 3 | 6 | 4.95% |
| G6PI_HUMAN | Glucose-6-phosphate isomerase | GPI | 63.1 | 100.0% | 2 | 3 | 5 | 5.73% |
| RS20_HUMAN | 40S ribosomal protein S20 | RPS20 | 13.4 | 100.0% | 3 | 3 | 5 | 19.30% |
| TCPD_HUMAN | T-complex protein 1 subunit delta | CCT4 | 57.9 | 100.0% | 2 | 2 | 4 | 5.38% |
| COF1_HUMAN | Cofilin-1 | CFL1 | 18.5 | 100.0% | 2 | 2 | 4 | 25.30% |
| RL27A_HUMAN | 60S ribosomal protein L27a | RPL27A | 16.6 | 100.0% | 2 | 2 | 4 | 16.20% |
| TGM3_HUMAN | Protein-glutamine gamma-glutamyltransferase E | TGM3 | 76.6 | 100.0% | 2 | 2 | 4 | 3.61% |
| CATA_HUMAN | Catalase | CAT | 59.8 | 100.0% | 2 | 2 | 4 | 4.17% |
| RS30_HUMAN | 40S ribosomal protein S30 | FAU | 6.6 | 100.0% | 2 | 2 | 4 | 18.60% |
| TGM1_HUMAN | Protein-glutamine gamma-glutamyltransferase K | TGM1 | 89.8 | 100.0% | 3 | 3 | 4 | 4.41% |
| RS4X_HUMAN | 40S ribosomal protein S4, X isoform | RPS4X | 29.6 | 100.0% | 11 | 12 | 43 | 41.40% |
| FLNA_HUMAN | Filamin-A | FLNA | 280.7 | 100.0% | 25 | 25 | 61 | 13.30% |
| FLNB_HUMAN | Filamin-B | FLNB | 278.2 | 100.0% | 14 | 14 | 24 | 8.69% |
| RS19_HUMAN | 40S ribosomal protein S19 | RPS19 | 16.1 | 100.0% | 10 | 10 | 17 | 57.20% |
| TCPG_HUMAN | T-complex protein 1 subunit gamma | CCT3 | 60.5 | 100.0% | 2 | 2 | 3 | 3.85% |
| EIF3B_HUMAN | Eukaryotic translation initiation factor 3 subunit B | EIF3B | 92.5 | 100.0% | 2 | 2 | 3 | 3.32% |
| RL17_HUMAN | 60S ribosomal protein L17 | RPL17 | 21.4 | 100.0% | 2 | 2 | 3 | 11.40% |
| 1433E_HUMAN | 14-3-3 protein epsilon | YWHAE | 29.2 | 100.0% | 2 | 2 | 3 | 9.41% |
| ACLY_HUMAN | ATP-citrate synthase | ACLY | 120.8 | 100.0% | 2 | 2 | 3 | 2.09% |
| GBLP_HUMAN | Guanine nucleotide-binding protein subunit beta-2-like 1 | GNB2L1 | 35.1 | 100.0% | 2 | 2 | 3 | 8.83% |
| SPB12_HUMAN | Serpin B12 | SERPINB12 | 46.3 | 100.0% | 2 | 2 | 3 | 6.42% |
| DESP_HUMAN | Desmoplakin | DSP | 331.8 | 100.0% | 54 | 59 | 123 | 21.90% |
| RS2_HUMAN | 40S ribosomal protein S2 | RPS2 | 31.3 | 100.0% | 7 | 8 | 29 | 26.30% |
| PRDX1_HUMAN | Peroxiredoxin-1 | PRDX1 | 22.1 | 100.0% | 5 | 6 | 14 | 26.10% |
| RS16_HUMAN | 40S ribosomal protein S16 | RPS16 | 16.4 | 100.0% | 9 | 11 | 17 | 47.30% |
| RS3A_HUMAN | 40S ribosomal protein S3a | RPS3A | 29.9 | 100.0% | 14 | 16 | 29 | 45.10% |
| ARGI1_HUMAN | Arginase-1 | ARG1 | 34.7 | 100.0% | 4 | 4 | 8 | 13.70% |
| TBB4B_HUMAN | Tubulin beta-4B chain | TUBB4B | 49.8 | 100.0% | 5 | 8 | 34 | 47.90% |
| TCPA_HUMAN | T-complex protein 1 subunit alpha | TCP1 | 60.3 | 100.0% | 2 | 2 | 2 | 3.60% |
| FILA_HUMAN | Filaggrin | FLG | 435.1 | 100.0% | 2 | 2 | 2 | 0.54% |
| XP32_HUMAN | Skin-specific protein 32 | XP32 | 26.2 | 99.8% | 2 | 2 | 2 | 8.00% |
| HMGB1_HUMAN | High mobility group protein B1 | HMGB1 | 24.9 | 100.0% | 2 | 2 | 2 | 14.40% |
| CYTS_HUMAN | Cystatin-S | CST4 | 16.2 | 100.0% | 2 | 2 | 2 | 13.50% |
| HSP74_HUMAN | Heat shock 70 kDa protein 4 | HSPA4 | 94.3 | 100.0% | 2 | 2 | 2 | 3.21% |
| IGHA1_HUMAN | Ig alpha-1 chain C region | IGHA1 | 37.7 | 100.0% | 2 | 2 | 2 | 5.10% |
| RS17L_HUMAN | 40S ribosomal protein S17-like | RPS17L | 15.6 | 100.0% | 8 | 9 | 14 | 71.90% |
| PLAK_HUMAN | Junction plakoglobin | JUP | 81.7 | 100.0% | 13 | 16 | 41 | 23.60% |
| RS9_HUMAN | 40S ribosomal protein S9 | RPS9 | 22.6 | 100.0% | 5 | 5 | 10 | 19.10% |
| FINC_HUMAN | Fibronectin | FN1 | 262.6 | 100.0% | 9 | 10 | 20 | 5.36% |
| RS5_HUMAN | 40S ribosomal protein S5 | RPS5 | 22.9 | 100.0% | 6 | 7 | 17 | 27.00% |
| RS15A_HUMAN | 40S ribosomal protein S15a | RPS15A | 14.8 | 100.0% | 5 | 5 | 8 | 47.70% |
| RL9_HUMAN | 60S ribosomal protein L9 | RPL9 | 21.9 | 100.0% | 7 | 8 | 15 | 30.70% |
| DSG1_HUMAN | Desmoglein-1 | DSG1 | 113.7 | 100.0% | 11 | 15 | 49 | 13.20% |
| DDX5_HUMAN | Probable ATP-dependent RNA helicase DDX5 | DDX5 | 69.1 | 100.0% | 6 | 6 | 11 | 11.70% |
| RL23A_HUMAN | 60S ribosomal protein L23a | RPL23A | 17.7 | 100.0% | 7 | 7 | 13 | 37.20% |
| RS7_HUMAN | 40S ribosomal protein S7 | RPS7 | 22.1 | 100.0% | 6 | 6 | 12 | 30.40% |
| IMB1_HUMAN | Importin subunit beta-1 | KPNB1 | 97.2 | 100.0% | 3 | 3 | 6 | 4.79% |
| TCPQ_HUMAN | T-complex protein 1 subunit theta | CCT8 | 59.6 | 100.0% | 2 | 2 | 4 | 5.29% |
| RS23_HUMAN | 40S ribosomal protein S23 | RPS23 | 15.8 | 100.0% | 4 | 4 | 8 | 28.70% |
| RAN_HUMAN | GTP-binding nuclear protein Ran | RAN | 24.4 | 100.0% | 3 | 3 | 4 | 16.20% |
| RS25_HUMAN | 40S ribosomal protein S25 | RPS25 | 13.7 | 100.0% | 6 | 6 | 14 | 41.60% |
| RS3_HUMAN | 40S ribosomal protein S3 | RPS3 | 26.7 | 100.0% | 8 | 9 | 31 | 36.20% |
| Mitochondrial fraction | | | | | | | | |
| LPP2_HUMAN | Lipid phosphate phosphohydrolase 2 | PPAP2C | 32.6 | 100.0% | 2 | 2 | 33 | 7.64% |
| APOB_HUMAN | Apolipoprotein B-100 | APOB | 515.6 | 100.0% | 4 | 4 | 29 | 1.16% |
| MYO1B_HUMAN | Unconventional myosin-Ib | MYO1B | 132.0 | 100.0% | 15 | 16 | 29 | 15.80% |
| VPP2_HUMAN | V-type proton ATPase 116 kDa subunit a isoform 2 | ATP6V0A2 | 98.1 | 100.0% | 9 | 10 | 24 | 14.70% |
| ODB2_HUMAN | Lipoamide acyltransferase component of branched-chain alpha-keto acid dehydrogenase complex, mitochondrial | DBT | 53.5 | 100.0% | 10 | 13 | 24 | 24.30% |
| SUN2_HUMAN | SUN domain-containing protein 2 | SUN2 | 80.3 | 100.0% | 10 | 10 | 23 | 18.30% |
| MIRO2_HUMAN | Mitochondrial Rho GTPase 2 | RHOT2 | 68.1 | 100.0% | 7 | 7 | 23 | 13.30% |
| RT09_HUMAN | 28S ribosomal protein S9, mitochondrial | MRPS9 | 45.8 | 100.0% | 10 | 12 | 22 | 27.00% |
| OXA1L_HUMAN | Mitochondrial inner membrane protein OXA1L | OXA1L | 48.5 | 100.0% | 7 | 9 | 22 | 17.50% |
| VTNC_HUMAN | Vitronectin | VTN | 54.3 | 100.0% | 2 | 2 | 22 | 4.81% |
| GNAI2_HUMAN | Guanine nucleotide-binding protein G(i) subunit alpha-2 | GNAI2 | 40.5 | 100.0% | 5 | 7 | 21 | 26.50% |
| CMC1_HUMAN | Calcium-binding mitochondrial carrier protein Aralar1 | SLC25A12 | 74.8 | 100.0% | 10 | 10 | 20 | 26.70% |
| VAT1_HUMAN | Synaptic vesicle membrane protein VAT-1 homolog | VAT1 | 41.9 | 100.0% | 5 | 7 | 19 | 18.30% |
| ACAD9_HUMAN | Acyl-CoA dehydrogenase family member 9, mitochondrial | ACAD9 | 68.8 | 100.0% | 10 | 10 | 19 | 15.60% |
| DNJA3_HUMAN | DnaJ homolog subfamily A member 3, mitochondrial | DNAJA3 | 52.5 | 100.0% | 6 | 8 | 19 | 17.10% |
| PLXB2_HUMAN | Plexin-B2 | PLXNB2 | 205.1 | 100.0% | 10 | 11 | 18 | 7.78% |
| AFG32_HUMAN | AFG3-like protein 2 | AFG3L2 | 88.6 | 100.0% | 12 | 13 | 18 | 16.80% |
| SUCB1_HUMAN | Succinyl-CoA ligase [ADP-forming] subunit beta, mitochondrial | SUCLA2 | 50.3 | 100.0% | 6 | 6 | 17 | 13.00% |
| IDH3B_HUMAN | Isocitrate dehydrogenase [NAD] subunit beta, mitochondrial | IDH3B | 42.2 | 100.0% | 8 | 9 | 16 | 19.70% |
| RL6_HUMAN | 60S ribosomal protein L6 | RPL6 | 32.7 | 100.0% | 5 | 6 | 15 | 19.80% |
| XRCC5_HUMAN | X-ray repair cross-complementing protein 5 | XRCC5 | 82.7 | 100.0% | 8 | 9 | 15 | 11.70% |
| OPA1_HUMAN | Dynamin-like 120 kDa protein, mitochondrial | OPA1 | 111.6 | 100.0% | 7 | 7 | 15 | 8.75% |
| NDUV1_HUMAN | NADH dehydrogenase [ubiquinone] flavoprotein 1, mitochondrial | NDUFV1 | 50.8 | 100.0% | 5 | 5 | 14 | 11.90% |
| CLPX_HUMAN | ATP-dependent Clp protease ATP-binding subunit clpX-like, mitochondrial | CLPX | 69.2 | 100.0% | 8 | 8 | 14 | 16.10% |
| COQ6_HUMAN | Ubiquinone biosynthesis monooxygenase COQ6, mitochondrial | COQ6 | 50.9 | 100.0% | 2 | 2 | 13 | 4.06% |
| RT02_HUMAN | 28S ribosomal protein S2, mitochondrial | MRPS2 | 33.3 | 100.0% | 5 | 5 | 13 | 12.80% |
| SUCA_HUMAN | Succinyl-CoA ligase [ADP/GDP-forming] subunit alpha, mitochondrial | SUCLG1 | 36.3 | 100.0% | 3 | 3 | 13 | 10.70% |
| MRP1_HUMAN | Multidrug resistance-associated protein 1 | ABCC1 | 171.6 | 100.0% | 8 | 8 | 13 | 6.99% |
| NRCAM_HUMAN | Neuronal cell adhesion molecule | NRCAM | 143.9 | 100.0% | 7 | 7 | 13 | 7.44% |
| ADAS_HUMAN | Alkyldihydroxyacetonephosphate synthase, peroxisomal | AGPS | 72.9 | 100.0% | 6 | 7 | 13 | 12.60% |
| AL7A1_HUMAN | Alpha-aminoadipicsemialdehyde dehydrogenase | ALDH7A1 | 58.5 | 100.0% | 8 | 8 | 13 | 17.30% |
| P5CS_HUMAN | Delta-1-pyrroline-5-carboxylate synthase | ALDH18A1 | 87.3 | 100.0% | 19 | 23 | 47 | 26.80% |
| CTRO_HUMAN | Citron Rho-interacting kinase | CIT | 231.4 | 100.0% | 6 | 6 | 12 | 3.31% |
| STT3A_HUMAN | Dolichyl-diphosphooligosaccharide--protein glycosyltransferase subunit STT3A | STT3A | 80.5 | 100.0% | 3 | 4 | 12 | 4.40% |
| IDH3A_HUMAN | Isocitrate dehydrogenase [NAD] subunit alpha, mitochondrial | IDH3A | 39.6 | 100.0% | 5 | 5 | 12 | 12.80% |
| MTCH1_HUMAN | Mitochondrial carrier homolog 1 | MTCH1 | 41.5 | 100.0% | 2 | 3 | 12 | 5.40% |
| DIC_HUMAN | Mitochondrial dicarboxylate carrier | SLC25A10 | 31.3 | 100.0% | 4 | 5 | 12 | 18.50% |
| PREP_HUMAN | Presequence protease, mitochondrial | PITRM1 | 117.4 | 100.0% | 7 | 8 | 11 | 9.45% |
| ACADM_HUMAN | Medium-chain specific acyl-CoA dehydrogenase, mitochondrial | ACADM | 46.6 | 100.0% | 6 | 6 | 11 | 15.70% |
| S35B2_HUMAN | Adenosine 3'-phospho 5'-phosphosulfate transporter 1 | SLC35B2 | 47.5 | 100.0% | 3 | 3 | 11 | 7.64% |
| KAD2_HUMAN | Adenylate kinase 2, mitochondrial | AK2 | 26.5 | 100.0% | 5 | 6 | 11 | 18.80% |
| LDLR_HUMAN | Low-density lipoprotein receptor | LDLR | 95.4 | 100.0% | 5 | 5 | 11 | 6.86% |
| MBOA7_HUMAN | Lysophospholipid acyltransferase 7 | MBOAT7 | 52.8 | 100.0% | 4 | 4 | 11 | 11.20% |
| ETFB_HUMAN | Electron transfer flavoprotein subunit beta | ETFB | 27.8 | 100.0% | 5 | 5 | 11 | 20.00% |
| AT2A2_HUMAN | Sarcoplasmic/endoplasmic reticulum calcium ATPase 2 | ATP2A2 | 114.8 | 100.0% | 6 | 6 | 10 | 7.10% |
| ABCB7_HUMAN | ATP-binding cassette sub-family B member 7, mitochondrial | ABCB7 | 82.6 | 100.0% | 7 | 7 | 10 | 11.40% |
| SCRB2_HUMAN | Lysosome membrane protein 2 | SCARB2 | 54.3 | 100.0% | 4 | 5 | 10 | 7.32% |
| EFGM_HUMAN | Elongation factor G, mitochondrial | GFM1 | 83.5 | 100.0% | 5 | 5 | 10 | 7.06% |
| S38A2_HUMAN | Sodium-coupled neutral amino acid transporter 2 | SLC38A2 | 56.0 | 100.0% | 4 | 4 | 10 | 9.68% |
| KAD4_HUMAN | Adenylate kinase 4, mitochondrial | AK4 | 25.3 | 100.0% | 5 | 6 | 10 | 26.90% |
| TBRG4_HUMAN | Protein TBRG4 | TBRG4 | 70.7 | 100.0% | 5 | 5 | 10 | 10.30% |
| CPT1A_HUMAN | Carnitine O-palmitoyltransferase 1, liver isoform | CPT1A | 88.4 | 100.0% | 4 | 4 | 10 | 6.34% |
| AL1B1_HUMAN | Aldehyde dehydrogenase X, mitochondrial | ALDH1B1 | 57.2 | 100.0% | 5 | 5 | 10 | 14.10% |
| LAT1_HUMAN | Large neutral amino acids transporter small subunit 1 | SLC7A5 | 55.0 | 100.0% | 2 | 2 | 9 | 6.31% |
| KDIS_HUMAN | Kinase D-interacting substrate of 220 kDa | KIDINS220 | 196.5 | 100.0% | 3 | 3 | 9 | 2.43% |
| STX4_HUMAN | Syntaxin-4 | STX4 | 34.2 | 100.0% | 4 | 4 | 9 | 16.20% |
| RT05_HUMAN | 28S ribosomal protein S5, mitochondrial | MRPS5 | 48.0 | 100.0% | 6 | 6 | 9 | 16.50% |
| MTX1_HUMAN | Metaxin-1 | MTX1 | 51.5 | 100.0% | 4 | 4 | 9 | 7.51% |
| RM15_HUMAN | 39S ribosomal protein L15, mitochondrial | MRPL15 | 33.4 | 100.0% | 3 | 3 | 9 | 11.80% |
| ECE1_HUMAN | Endothelin-converting enzyme 1 | ECE1 | 87.2 | 100.0% | 2 | 2 | 9 | 4.55% |
| NDUB5_HUMAN | NADH dehydrogenase [ubiquinone] 1 beta subcomplex subunit 5, mitochondrial | NDUFB5 | 21.8 | 100.0% | 4 | 5 | 9 | 29.60% |
| ABCBA_HUMAN | ATP-binding cassette sub-family B member 10, mitochondrial | ABCB10 | 79.2 | 100.0% | 6 | 6 | 9 | 9.76% |
| ATD3B_HUMAN | ATPase family AAA domain-containing protein 3B | ATAD3B | 72.6 | 100.0% | 5 | 6 | 9 | 34.10% |
| 1A34_HUMAN | HLA class I histocompatibility antigen, A-34 alpha chain | HLA-A | 41.1 | 100.0% | 3 | 3 | 9 | 34.50% |
| XPP3_HUMAN | Probable Xaa-Pro aminopeptidase 3 | XPNPEP3 | 57.0 | 100.0% | 4 | 5 | 9 | 9.86% |
| BCS1_HUMAN | Mitochondrial chaperone BCS1 | BCS1L | 47.5 | 100.0% | 5 | 5 | 9 | 12.90% |
| HMOX1_HUMAN | Heme oxygenase 1 | HMOX1 | 32.8 | 100.0% | 4 | 4 | 9 | 13.50% |
| G6PI_HUMAN | Glucose-6-phosphate isomerase | GPI | 63.1 | 100.0% | 3 | 4 | 8 | 7.35% |
| FAKD2_HUMAN | FAST kinase domain-containing protein 2 | FASTKD2 | 81.5 | 100.0% | 7 | 7 | 8 | 12.00% |
| APMAP_HUMAN | Adipocyte plasma membrane-associated protein | APMAP | 46.5 | 100.0% | 5 | 6 | 8 | 12.70% |
| ECI2_HUMAN | Enoyl-CoA delta isomerase 2, mitochondrial | ECI2 | 43.6 | 100.0% | 4 | 6 | 8 | 13.20% |
| FMNL2_HUMAN | Formin-like protein 2 | FMNL2 | 123.3 | 100.0% | 5 | 5 | 8 | 5.16% |
| NU4M_HUMAN | NADH-ubiquinone oxidoreductase chain 4 | MT-ND4 | 51.6 | 100.0% | 2 | 2 | 8 | 4.14% |
| MUTA_HUMAN | Methylmalonyl-CoA mutase, mitochondrial | MUT | 83.1 | 100.0% | 5 | 5 | 8 | 7.87% |
| ACOT1_HUMAN | Acyl-coenzyme A thioesterase 1 | ACOT1 | 46.3 | 100.0% | 4 | 4 | 8 | 9.74% |
| CPT2_HUMAN | Carnitine O-palmitoyltransferase 2, mitochondrial | CPT2 | 73.8 | 100.0% | 4 | 4 | 8 | 6.69% |
| NCEH1_HUMAN | Neutral cholesterol ester hydrolase 1 | NCEH1 | 45.8 | 100.0% | 4 | 4 | 8 | 13.00% |
| SURF4_HUMAN | Surfeit locus protein 4 | SURF4 | 30.4 | 100.0% | 3 | 4 | 8 | 15.20% |
| ECI1_HUMAN | Enoyl-CoA delta isomerase 1, mitochondrial | ECI1 | 32.8 | 100.0% | 3 | 4 | 8 | 11.90% |
| Nuclear fraction | | | | | | | | |
| PLEC_HUMAN | Plectin | PLEC | 531.8 | 100.0% | 98 | 103 | 224 | 24.40% |
| PRP8_HUMAN | Pre-mRNA-processing-splicing factor 8 | PRPF8 | 273.6 | 100.0% | 29 | 29 | 51 | 15.50% |
| U520_HUMAN | U5 small nuclear ribonucleoprotein 200 kDa helicase | SNRNP200 | 244.5 | 100.0% | 28 | 31 | 51 | 17.60% |
| MATR3_HUMAN | Matrin-3 | MATR3 | 94.6 | 100.0% | 16 | 17 | 50 | 23.10% |
| ILF3_HUMAN | Interleukin enhancer-binding factor 3 | ILF3 | 95.3 | 100.0% | 19 | 22 | 49 | 24.70% |
| HNRL2_HUMAN | Heterogeneous nuclear ribonucleoprotein U-like protein 2 | HNRNPUL2 | 85.1 | 100.0% | 14 | 17 | 34 | 18.50% |
| U5S1_HUMAN | 116 kDa U5 small nuclear ribonucleoprotein component | EFTUD2 | 109.4 | 100.0% | 15 | 16 | 29 | 19.40% |
| ROA3_HUMAN | Heterogeneous nuclear ribonucleoprotein A3 | HNRNPA3 | 39.6 | 100.0% | 5 | 9 | 29 | 16.40% |
| RL6_HUMAN | 60S ribosomal protein L6 | RPL6 | 32.7 | 100.0% | 7 | 8 | 28 | 22.60% |
| NOP56_HUMAN | Nucleolar protein 56 | NOP56 | 66.1 | 100.0% | 13 | 14 | 28 | 27.90% |
| RALY_HUMAN | RNA-binding protein Raly | RALY | 32.5 | 100.0% | 8 | 9 | 28 | 28.10% |
| FILA_HUMAN | Filaggrin | FLG | 435.1 | 100.0% | 13 | 14 | 26 | 4.51% |
| SRSF6_HUMAN | Serine/arginine-rich splicing factor 6 | SRSF6 | 39.6 | 100.0% | 3 | 3 | 23 | 9.30% |
| PININ_HUMAN | Pinin | PNN | 81.6 | 100.0% | 9 | 10 | 23 | 13.80% |
| RL1D1_HUMAN | Ribosomal L1 domain-containing protein 1 | RSL1D1 | 55.0 | 100.0% | 13 | 13 | 23 | 23.30% |
| SRSF1_HUMAN | Serine/arginine-rich splicing factor 1 | SRSF1 | 27.7 | 100.0% | 9 | 10 | 23 | 28.60% |
| DDX3X_HUMAN | ATP-dependent RNA helicase DDX3X | DDX3X | 73.2 | 100.0% | 5 | 6 | 20 | 10.40% |
| SBSN_HUMAN | Suprabasin | SBSN | 60.5 | 100.0% | 4 | 4 | 19 | 11.50% |
| NOP2_HUMAN | Probable 28S rRNA (cytosine(4447)-C(5))-methyltransferase | NOP2 | 89.3 | 100.0% | 10 | 10 | 19 | 14.40% |
| NONO_HUMAN | Non-POU domain-containing octamer-binding protein | NONO | 54.2 | 100.0% | 7 | 8 | 17 | 19.70% |
| HNRL1_HUMAN | Heterogeneous nuclear ribonucleoprotein U-like protein 1 | HNRNPUL1 | 95.7 | 100.0% | 8 | 9 | 16 | 12.60% |
| RB12B_HUMAN | RNA-binding protein 12B | RBM12B | 118.1 | 100.0% | 7 | 7 | 16 | 7.39% |
| HNRDL_HUMAN | Heterogeneous nuclear ribonucleoprotein D-like | HNRNPDL | 46.4 | 100.0% | 5 | 7 | 16 | 11.40% |
| HP1B3_HUMAN | Heterochromatin protein 1-binding protein 3 | HP1BP3 | 61.2 | 100.0% | 7 | 7 | 15 | 8.86% |
| ROAA_HUMAN | Heterogeneous nuclear ribonucleoprotein A/B | HNRNPAB | 36.2 | 100.0% | 4 | 5 | 15 | 13.00% |
| DDX21_HUMAN | Nucleolar RNA helicase 2 | DDX21 | 87.3 | 100.0% | 23 | 25 | 80 | 32.70% |
| IF2B1_HUMAN | Insulin-like growth factor 2 mRNA-binding protein 1 | IGF2BP1 | 63.5 | 100.0% | 6 | 7 | 14 | 12.30% |
| SRSF7_HUMAN | Serine/arginine-rich splicing factor 7 | SRSF7 | 27.4 | 100.0% | 6 | 7 | 14 | 23.50% |
| DDX17_HUMAN | Probable ATP-dependent RNA helicase DDX17 | DDX17 | 80.3 | 100.0% | 6 | 7 | 14 | 19.20% |
| DDX27_HUMAN | Probable ATP-dependent RNA helicase DDX27 | DDX27 | 89.8 | 100.0% | 7 | 7 | 13 | 9.55% |
| NOG1_HUMAN | Nucleolar GTP-binding protein 1 | GTPBP4 | 74.0 | 100.0% | 4 | 4 | 13 | 6.94% |
| PESC_HUMAN | Pescadillo homolog | PES1 | 68.0 | 100.0% | 7 | 8 | 13 | 12.20% |
| NOC3L_HUMAN | Nucleolar complex protein 3 homolog | NOC3L | 92.6 | 100.0% | 6 | 6 | 13 | 8.50% |
| DHX15_HUMAN | Pre-mRNA-splicing factor ATP-dependent RNA helicase DHX15 | DHX15 | 90.9 | 100.0% | 7 | 7 | 13 | 10.30% |
| NOP58_HUMAN | Nucleolar protein 58 | NOP58 | 59.6 | 100.0% | 7 | 9 | 13 | 15.90% |
| RBM14_HUMAN | RNA-binding protein 14 | RBM14 | 69.5 | 100.0% | 8 | 8 | 13 | 14.10% |
| ZN326_HUMAN | DBIRD complex subunit ZNF326 | ZNF326 | 65.7 | 100.0% | 7 | 7 | 13 | 12.50% |
| TRI27_HUMAN | Zinc finger protein RFP | TRIM27 | 58.5 | 100.0% | 7 | 7 | 12 | 18.90% |
| HNRPQ_HUMAN | Heterogeneous nuclear ribonucleoprotein Q | SYNCRIP | 69.6 | 100.0% | 5 | 5 | 12 | 18.30% |
| RBP56_HUMAN | TATA-binding protein-associated factor 2N | TAF15 | 61.8 | 100.0% | 4 | 4 | 12 | 11.00% |
| FBRL_HUMAN | rRNA 2'-O-methyltransferase fibrillarin | FBL | 33.8 | 100.0% | 6 | 6 | 11 | 26.80% |
| ACINU_HUMAN | Apoptotic chromatin condensation inducer in the nucleus | ACIN1 | 151.9 | 100.0% | 4 | 4 | 11 | 3.50% |
| PRP19_HUMAN | Pre-mRNA-processing factor 19 | PRPF19 | 55.2 | 100.0% | 7 | 7 | 11 | 19.00% |
| ROA0_HUMAN | Heterogeneous nuclear ribonucleoprotein A0 | HNRNPA0 | 30.8 | 100.0% | 6 | 7 | 11 | 24.30% |
| CDC5L_HUMAN | Cell division cycle 5-like protein | CDC5L | 92.3 | 100.0% | 5 | 5 | 10 | 6.61% |
| CASPE_HUMAN | Caspase-14 | CASP14 | 27.7 | 100.0% | 4 | 5 | 10 | 15.70% |
| H10_HUMAN | Histone H1.0 | H1F0 | 20.9 | 100.0% | 4 | 5 | 10 | 23.20% |
| TRA2A_HUMAN | Transformer-2 protein homolog alpha | TRA2A | 32.7 | 100.0% | 4 | 4 | 10 | 12.40% |
| FUBP1_HUMAN | Far upstream element-binding protein 1 | FUBP1 | 67.6 | 100.0% | 6 | 6 | 10 | 11.20% |
| RL35A_HUMAN | 60S ribosomal protein L35a | RPL35A | 12.5 | 100.0% | 4 | 5 | 9 | 34.50% |
| NFL_HUMAN | Neurofilament light polypeptide | NEFL | 61.5 | 100.0% | 6 | 6 | 9 | 14.40% |
| HNRPM_HUMAN | Heterogeneous nuclear ribonucleoprotein M | HNRNPM | 77.5 | 100.0% | 24 | 32 | 135 | 41.20% |
| DSC1_HUMAN | Desmocollin-1 | DSC1 | 100.0 | 100.0% | 2 | 2 | 8 | 2.57% |
| SAP18_HUMAN | Histone deacetylase complex subunit SAP18 | SAP18 | 17.6 | 100.0% | 3 | 5 | 8 | 15.70% |
| RNPS1_HUMAN | RNA-binding protein with serine-rich domain 1 | RNPS1 | 34.2 | 100.0% | 2 | 2 | 8 | 7.54% |
| RPF2_HUMAN | Ribosome production factor 2 homolog | RPF2 | 35.6 | 100.0% | 4 | 5 | 8 | 12.70% |
| PABP2_HUMAN | Polyadenylate-binding protein 2 | PABPN1 | 32.7 | 100.0% | 3 | 3 | 8 | 12.40% |
| FUBP2_HUMAN | Far upstream element-binding protein 2 | KHSRP | 73.1 | 100.0% | 3 | 3 | 8 | 5.20% |
| DHX9_HUMAN | ATP-dependent RNA helicase A | DHX9 | 141.0 | 100.0% | 24 | 28 | 73 | 22.00% |
| PCBP1_HUMAN | Poly(rC)-binding protein 1 | PCBP1 | 37.5 | 100.0% | 4 | 4 | 7 | 15.70% |
| RL21_HUMAN | 60S ribosomal protein L21 | RPL21 | 18.6 | 100.0% | 3 | 4 | 7 | 27.50% |
| SNW1_HUMAN | SNW domain-containing protein 1 | SNW1 | 61.5 | 100.0% | 4 | 4 | 7 | 8.77% |
| DKC1_HUMAN | H/ACA ribonucleoprotein complex subunit 4 | DKC1 | 57.7 | 100.0% | 4 | 4 | 7 | 10.90% |
| PRP6_HUMAN | Pre-mRNA-processing factor 6 | PRPF6 | 106.9 | 100.0% | 4 | 4 | 7 | 4.89% |
| UBF1_HUMAN | Nucleolar transcription factor 1 | UBTF | 89.4 | 100.0% | 4 | 4 | 7 | 5.76% |
| TOP2A_HUMAN | DNA topoisomerase 2-alpha | TOP2A | 174.4 | 100.0% | 6 | 6 | 7 | 4.77% |
| SRSF9_HUMAN | Serine/arginine-rich splicing factor 9 | SRSF9 | 25.5 | 100.0% | 4 | 4 | 7 | 19.50% |

**Supplementary Table2. Characteristics of 15NSCLC patients**

| **Characteristics** | **No. of patients** |
| --- | --- |
| **Age (years)** |  |
| ≤ 60 | 4 |
| ＞ 60 | 11 |
| **Gender** |  |
| Female | 5 |
| Male | 10 |
| **Tumor type** |  |
| Adenocarcinoma | 14 |
| Pleomorphic carcinoma | 1 |
| **Overall stage** |  |
| 1-2 | 12 |
| 3 | 3 |
| **Tumor grade** |  |
| I-II | 13 |
| III-IV | 2 |

**Supplementary Table 3.Immunohistochemicalanalysis of hnRNP A3 and EGFR expression in 15 paired tumors and adjacent normal tissues of NSCLC.**

|  | Percentage of hnRNP A3 positive staining (%)* | | | | Percentage of EGFR positive staining (%)* | | | |
| --- | --- | --- | --- | --- | --- | --- | --- | --- |
|  | Negative  (0%) | Low  (1-50%) | Medium  (51-95%) | High  (96-100%) | Negative  (0%) | Low  (1-50%) | Medium  (51-95%) | High  (96-100%) |
| Adjacent normal tissue (N=15) | 0 | 0 | 8 | 7 | 0 | 0 | 15 | 0 |
| Tumor (N=15) | 0 | 0 | 0 | 15 | 0 | 0 | 1 | 14 |

* The percentage of positive staining cells was determined in a representative large section of each tissue specimen. The immunoreactivity was grouped into four groups according to the percentage of the positive tumor cells: negative (0%), low (1-50%), medium (51-95%), high (96-100%).
