# Supplementary figures and images for: Profiling of subcellular EGFR interactome reveals hnRNP A3 modulates nuclear EGFR localization
Source: Oncogenesis. 2020 Apr 22;9(4):40. doi: 10.1038/s41389-020-0225-0 (PMC7176650; doi:10.1038/s41389-020-0225-0)

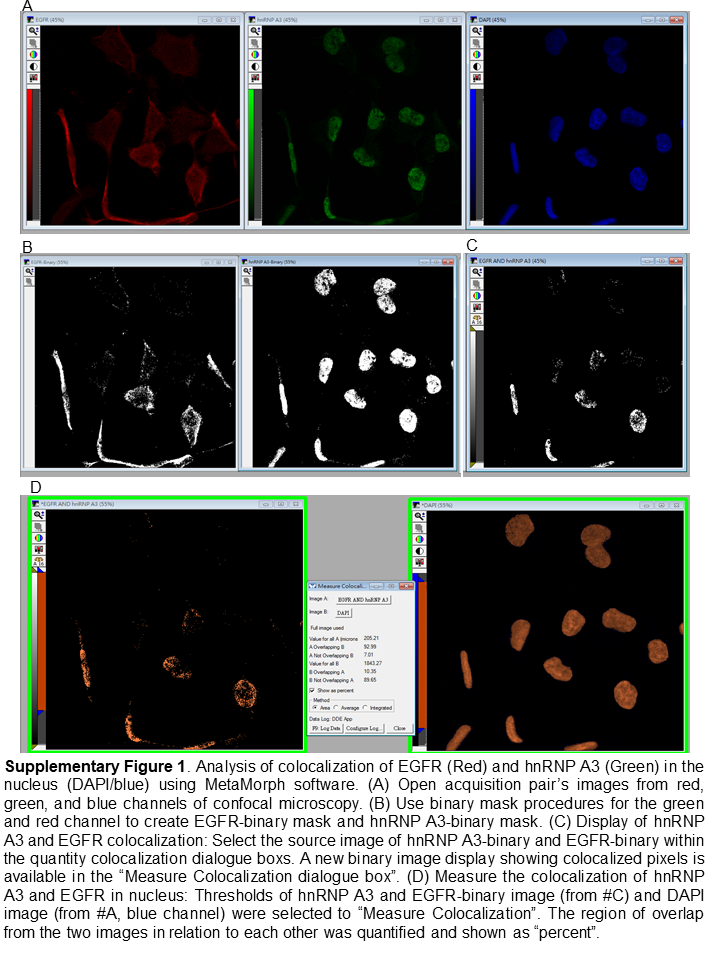

Supplement: Supplementary file 2 — Supplementary Figure 1 [file 41389_2020_225_MOESM2_ESM.tif]
